# Population‐specific validation of long bone linear discriminant analysis against morphoscopic pelvic methods for sex estimation in contemporary Italian skeletal remains

**DOI:** 10.1111/1556-4029.70293

**Published:** 2026-02-28

**Authors:** Siam Knecht, Paolo Morandini, Lucie Biehler‐Gomez, Mustapha Ouladsine, Christophe Roman, Cristina Cattaneo, Pascal Adalian

**Affiliations:** ^1^ Aix Marseille Univ, CNRS, EFS, ADES Marseille France; ^2^ LABANOF (Laboratorio di Antropologia e Odontologia Forense), Department of Biomedical Science for Health University of Milan Milan Italy; ^3^ LIS UMR 7020 CNRS, Aix Marseille Univ Marseille France

**Keywords:** forensic anthropology, linear discriminant analysis, long bones, machine learning, population‐specific validation, sex estimation

## Abstract

Sex estimation represents a fundamental step in forensic identification protocols, traditionally relying on morphoscopic pelvic assessment. However, the increasing integration of machine learning approaches and population‐specific validation requirements necessitate comprehensive evaluation of alternative methodologies. This study provides the first direct comparison of established morphoscopic methods (Phenice 1969, Bruzek 2002) against a multivariable long bone linear discriminant analysis (LDA) model in a sample of 333 documented individuals from the CAL Milano Cemetery Skeletal Collection. Metric data were preprocessed to address missing values through imputation prior to analysis. The morphoscopic methods achieved high accuracy rates (Phenice: 96.9%, Bruzek: 97.7%) but showed significant exclusion rates due to preservation limitations (32.4% and 2.4% respectively). The long bone LDA model demonstrated comparable performance with threshold‐dependent accuracy ranging from 95.2% (0.50 threshold) to 98.1% (0.88 threshold), while maintaining universal applicability across all specimens. Crucially, disagreement analysis revealed method‐specific error patterns with minimal overlap in misclassified individuals, supporting complementary rather than redundant diagnostic signals. These findings validate long bones as a reliable alternative for sex estimation in fragmentary remains while establishing population‐specific accuracy benchmarks for contemporary Italian forensic applications. The threshold‐adjustable probabilistic framework offers operational flexibility for balancing classification certainty against sample coverage requirements.

**Highlights**

- Threshold‐adjustable probabilistic frameworks were developed for sex estimation.
- Morphoscopic and metric methods showed minimal misclassification.
- Long bone LDA performed best, reaching 98.5% accuracy while maintaining universal applicability.
- Preservation constraints limited applicability of Phenice 1969 and Bruzek 2002 despite >96% accuracy.

## 1 INTRODUCTION

Biological sex estimation constitutes a critical component of the forensic identification process, directly impacting the scope of potential matches in missing person databases [1, 2]. The pelvis remains the anatomical reference standard due to its dimorphism, with established morphoscopic methods achieving >95% accuracy in optimal preservation conditions [3, 4, 5]. However, contemporary forensic practice can be confronted with fragmentary remains where traditional pelvic assessment proves inadequate or impossible [6, 7].

Recent methodological advances in forensic anthropology have demonstrated the potential of machine learning approaches for sex estimation, with multivariable long bone models achieving 90–97% accuracy in cross‐validation studies [8, 9, 10]. Deep learning networks applied to cranial CT reconstructions report an accuracy of up to 97%, substantially outperforming results obtained by trained human assessors applying conventional morphoscopic methods [11]. These developments align with the broader integration of artificial intelligence in forensic sciences, where computational approaches can offer enhanced objectivity and reproducibility [12, 13].

Population variation has long been recognized as an important factor influencing the performance of sex estimation methods in forensic anthropology (e.g. Ref. [14]). Recent research has increasingly focused on validating existing approaches across diverse populations to assess their transferability and reliability [15, 16]. Cross‐population application of sex estimation methods typically yields 5%–15% accuracy reductions, with Italian‐specific validation studies demonstrating the necessity of population‐calibrated approaches [17, 18, 19]. The CAL Milano Cemetery Skeletal Collection, representing one of Europe's largest documented contemporary collections (circa 2100 individuals), provides an optimal framework for establishing population‐specific performance benchmarks, namely accuracy ranges reported in previous Italian validation studies (e.g., Ref. [18]) serving as comparative reference points for contemporary forensic applications [20].

Current forensic practice demands methodological approaches that balance classification accuracy with universal applicability. While morphoscopic pelvic methods excel when preservation permits assessment, their applicability diminishes substantially in fragmentary contexts [21, 22]. Long bones, due to their structural robustness, are frequently recovered in better preservation states, making them attractive candidates for reliable sex estimation protocols [23].

In addition, recent studies have emphasized the importance of incorporating probability thresholds in discriminant and logistic models for sex estimation, as they enable the practitioner to control the trade‐off between classification confidence and rate of indeterminate estimations [24, 25]. Higher probability thresholds can improve certainty in classification outcomes but may also increase the number of indeterminate cases, thereby influencing the practical applicability of statistical methods in forensic settings.

This study addresses three critical questions in contemporary forensic sex estimation: (1) How do multivariable long bone models perform against established morphoscopic pelvic methods in the same population sample? (2) What degree of methodological complementarity exists between morphoscopic pelvic methods and metric approaches applied to long bones? (3) Can the use of probability thresholds enhance operational flexibility by allowing practitioners to balance classification confidence against the rate of indeterminate estimations for forensic applications?

We present the first comprehensive comparison of the Phenice 1969 [3] and Bruzek 2002 [4] morphoscopic methods against a validated long bone Linear Discriminant Analysis (LDA) model originally developed by Knecht et al. [8]. In this study, the existing and revised model was applied to the CAL Milano Cemetery Skeletal Collection to test its population‐specific performance and operational reliability in a contemporary Italian forensic context. This approach establishes population‐specific accuracy benchmarks and evaluates the external validity of a previously published discriminant framework.

## 2 MATERIALS AND METHODS

### 2.1 Sample composition

The study utilized 333 documented individuals from the CAL Milano Cemetery Skeletal Collection housed at the Laboratory of Forensic Anthropology and Odontology (LABANOF), University of Milan [20]. This contemporary collection includes circa 2100 unclaimed individuals with complete demographic documentation. The study sample comprised 161 males and 172 females (age range: 20–104 years, mean: 70.2 years, standard deviation: 17.1 years), with years of birth ranging from 1880 to 1972 and years of death from 1927 to 2001, selected based on adequate preservation of both pelvic elements and at least two long bones.

### 2.2 Morphoscopic pelvic assessment

Sex estimation from the pelvis employed two established morphoscopic protocols: Phenice 1969 [3] method evaluating three pubic traits (ventral arc, subpubic concavity, medial ischiopubic ramus aspect), and Bruzek 2002 [4] method assessing five pelvic morphological features (preauricular surface, greater sciatic notch, composite arch, inferior pelvis, ischiopubic proportion).

All assessments were conducted by a single experienced observer (PM) with 3 years of specialized training in morphoscopic sex estimation. For Phenice (1969), the final estimation was based on majority of female or male characteristics, consistent with validation protocols [26, 27]. Classification required observation of at least one trait. When only a single trait could be assessed, sex estimation was based on the result of that trait alone. In practice, nearly all cases had all three Phenice traits scored, with only three individuals presenting a single observable trait, the ventral arc, generally regarded as the most accurate of the triad (e.g., [21, 22, 26, 28]).

For Bruzek 2002, final classification required assessment of a minimum of two clearly observable traits, following the original methodology statement of applicability for fragmentary remains [4].

### 2.3 Long bones metric analysis

Sex estimation from long bones employed the validated multivariable approach of Knecht et al. [8], revised with 16 standardized osteometric variables across four long bones (humerus, radius, femur, tibia). Individuals were included when at least two long bones were available, ensuring a minimum of two measurable variables per individual. Although the number of recorded measurements varied due to preservation, cases with fewer than six measurements were extremely uncommon. Measurements followed Martin & Saller 1957 protocols [29] using digital calipers and an osteometric board, prioritizing left‐side elements with right‐side substitution when necessary.

Variables included maximum lengths, epiphyseal breadths, and mid‐shaft diameters, selected for documented sexual dimorphism and cross‐collection reliability [30, 31]. The complete list of anatomical variables is presented in Table 1. All measurements were performed by a single observer (SK) following Data Collection Procedures 2.0 standards for forensic osteometry [32].

**TABLE 1:** Long bones measurements selected for this study.

Anatomical localization	Bone	Code	Measurement
Upper limb	Humerus	humxln	Maximum length
		humebr	Distal epicondylar breadth
		humhhd	Maximum vertical diameter of the head
		hummxd	Maximum diameter midshaft
		hummwd	Minimum diameter midshaft
	Radius	radxln	Maximum length
		radtvd	Transverse diameter at midshaft
		radapd	Antero‐posterior diameter at midshaft
Lower limb	Femur	femxln	Maximum length
		fembln	Physiological length
		femebr	Epicondylar breadth
		femhhd	Maximum diameter of the head
		femmtv	Medio‐lateral diameter of the midshaft
		femmap	Antero‐posterior diameter of the midshaft
	Tibia	tibxln	Lateral condyle ‐ medial malleolus length
		tibpeb	Maximum proximal epiphyseal breadth

Prior to modeling, incomplete osteometric data were addressed to ensure analytical consistency. Missing values were estimated using an iterative regression imputation procedure [33], which predicts each missing entry based on the correlations observed among the remaining variables through successive modeling cycles.

This method was implemented in Python (version 3.11.7) using the IterativeImputer algorithm from the scikit‐learn library. Imputation was applied only to individuals with at least two measurable bones and a minimum of two directly observed variables. The proportion of imputed values represented less than 10% of the total dataset.

Imputation was used solely to explore model performance under conditions of partial data availability, and further studies are needed to determine whether such an approach could ever be appropriate or beneficial in real‐world forensic applications.

### 2.4 Reliability assessment

Measurement reliability evaluation included both intra‐ and inter‐observer components. For metric data, Technical Error of Measurement (TEM) and Intraclass Correlation Coefficient (ICC) quantified precision, with acceptable thresholds set at rTEM <5% and ICC >0.90 [31, 32]. For the morphoscopic methods, observer agreement was measured using the unweighted Cohen's Kappa coefficient [34] and the results were interpreted in accordance with established guidelines in the literature [35].

For morphoscopic sex estimation methods, observer agreement was assessed using Cohen's Kappa coefficient. Specifically, the Phenice 1969 method, which yields a binary nominal outcome (male/female), was evaluated using the unweighted Cohen's Kappa coefficient [34]. For the Bruzek 2002 method, the three possible outcomes (male–indeterminate–female) were treated as an ordinal scale, and observer agreement was therefore assessed using the weighted Cohen's Kappa [36]. The results were interpreted in accordance with established guidelines in the literature [35].

Regarding the metric analysis, intra‐observer reliability was based on repeated measurements by the same observer (SK) on the entire sample (*n* = 333) at 6‐month intervals. Inter‐observer evaluation involved independent assessment by two observers (SK and PM) on the entire sample (*n* = 333). To evaluate intra‐observer reliability of the Phenice 1969 and Bruzek 2002 methods, a random sample of 17 individuals was selected, and the same observer (PM) repeated the morphological assessments approximately 6 months later. For the inter‐observer analysis, 15 skeletons were randomly chosen and independently assessed by two observers (PM and LB‐G).

These statistical assessments were conducted to ensure the accuracy and reproducibility of the data, which are essential for the overall validity of the study findings.

### 2.5 Statistical framework and threshold implementation

All statistical analyses, including reliability tests (TEM and ICC) and descriptive statistics, were conducted in R (version 4.5.1) (library *irr*), while machine learning procedures and data preprocessing were performed in Python (version 3.11.7).

The long bone data underwent LDA using established protocols [8]. The LDA algorithm computed discriminant scores and output posterior probability of sex class, for each individual. Basically, the standard posterior probability is fixed at 0.50 and consequently assigns each individual to the most likely class (male or female), ensuring complete sample classification. However, this approach treats borderline probabilities (e.g., 0.51 vs. 0.99) as equivalent, potentially oversimplifying the uncertainty inherent in biological variation [37].

To address this limitation, we implemented multiple posterior probability thresholds (0.50, 0.70, and 0.88) to explicitly evaluate the trade‐off between accuracy and sample coverage. Lower thresholds (e.g., 0.50) maximize inclusion but include more uncertain cases, while higher thresholds exclude ambiguous classifications to enhance confidence in assigned sex. The thresholds of 0.70 and 0.88 were selected as illustrative examples that capture the range observed in our dataset: at 0.70, accuracy improved markedly with minimal data exclusion, whereas at 0.88, accuracy gains plateaued while the proportion of classified individuals decreased substantially (approximately 14%). For each threshold, sensitivity (male accuracy), specificity (female accuracy), and total accuracy (*n* correctly classified / (*n* correctly classified + *n* incorrectly classified)) were reported. Class discrimination bias, calculated as the difference between male (sensitivity) and female (specificity) accuracies, was considered acceptable within a 5% range [5].

This threshold‐adjustable design follows recent recommendations for probabilistic sex estimation models [24, 25] and provides a transparent framework for tailoring classification decisions to case‐specific reliability requirements.

### 2.6 Comparative analysis protocol

Method comparison evaluated accuracy, exclusion rates, and agreement patterns across all approaches. Agreement analysis examined cases where multiple methods could be applied, quantifying concordance rates and characterizing disagreement patterns. Particular attention focused on identifying method‐specific error patterns and assessing the potential for complementary diagnostic approaches.

## 3 RESULTS

### 3.1 Measurement reliability validation

Metric reliability assessment demonstrated high precision across all variables (Table 2). Inter‐observer analysis yielded ICC values exceeding 0.90 and rTEM values <5% for all measurements except humeral mid‐shaft maximum diameter (6.66%). Intra‐observer evaluation showed rTEM <4% and ICC approaching 1.0 for length measurements, confirming excellent measurement consistency.

**TABLE 2:** Intra and inter‐observer reliability for long bones measurements.

Measurements	Inter‐observer			Intra‐observer		
	TEM (mm)	rTEM	ICC	TEM (mm)	rTEM	ICC
humxln	1.43	0.46	0.99	0.76	0.23	0.99
humebr	0.60	1.03	0.99	0.29	0.50	0.99
humhhd	0.71	0.59	0.97	0.66	1.48	0.97
hummxd	1.36	6.66	0.90	0.64	3.10	0.94
hummwd	0.71	3.58	0.90	0.67	3.68	0.91
radxln	1.26	0.56	0.99	0.39	0.17	1.00
radtvd	0.56	3.84	0.91	0.38	2.58	0.96
radapd	0.44	3.96	0.90	0.39	3.52	0.92
femxln	1.52	0.35	0.99	0.34	0.08	1.00
fembln	1.40	0.32	0.99	0.41	0.10	1.00
femebr	0.76	0.97	0.99	0.47	0.61	0.99
femhhd	0.78	1.74	0.96	0.44	1.00	0.99
femmtv	0.66	2.45	0.94	0.42	1.53	0.97
femmap	0.64	2.33	0.99	0.54	1.95	0.97
tibxln	2.54	0.73	0.99	0.82	0.24	1.00
tibpeb	0.99	1.37	0.97	0.39	0.59	0.99

Morphoscopic reliability achieved substantial to perfect agreement levels (Table 3). The Phenice 1969 method demonstrated perfect intra‐ and inter‐observer concordance (κ = 1.000) across all traits. Bruzek 2002 method showed κ values ranging from 0.714 (substantial) to 1.000 (perfect), with final sex estimations achieving complete observer agreement in all evaluated cases.

**TABLE 3:** Intra‐ and inter‐observer agreement of morphological traits.

Method	Morphological feature	Intra‐observer			Inter‐observer		
		*n*	*K*	Agreement	*n*	*K*	Agreement
Phenice 1969	Ventral arc	14	1.000	Perfect	12	1.000	Perfect
	Subpubic concavity	14	1.000	Perfect	12	1.000	Perfect
	Medial aspect ischiopubic ramus	14	1.000	Perfect	12	1.000	Perfect
Bruzek 2002	Preauricular surface I	17	0.962	Almost perfect	11	1.000	Perfect
	Preauricular surface II	17	0.959	Almost perfect	11	1.000	Perfect
	Preauricular surface III	17	0.955	Almost perfect	11	0.935	Almost perfect
	Great sciatic notch I	17	1.000	Perfect	15	0.885	Almost perfect
	Great sciatic notch II	17	0.885	Almost perfect	15	0.885	Almost perfect
	Great sciatic notch III	17	1.000	Perfect	15	1.000	Perfect
	Composite arch	17	1.000	Perfect	15	0.963	Almost perfect
	Inferior pelvis I	13	1.000	Perfect	11	0.747	Substantial
	Inferior pelvis II	13	1.000	Perfect	11	0.940	Almost perfect
	Inferior pelvis III	13	1.000	Perfect	11	0.851	Almost perfect
	Ischiopubic proportion	13	1.000	Perfect	12	0.946	Almost perfect

*Note*: *n* = number of individuals; K = Cohen's Kappa (unweighted for Phenice 1969; weighted for Bruzek 2002); I = first condition; II = second condition; III = third condition.

### 3.2 Method performance comparison

#### 3.2.1 Morphoscopic methods

The Bruzek 2002 method achieved 97.7% accuracy (301/308 correctly classified) with a 2.4% exclusion rate due to insufficient preservation and a 5.1% indeterminate classifications (Table 4). The Phenice 1969 method demonstrated 96.9% accuracy (218/225 correctly classified) but exhibited substantial exclusion rates (32.4%) due to pubic region preservation requirements (Table 4).

**TABLE 4:** Results of sex estimation methods for the 333 individuals.

Total						
Method	Threshold	Correct (*n*)	Incorrect (*n*)	Excluded^a^ (%)	Indeterminate (%)^b^	Accuracy (%)
Bruzek 2002	‐	301	7	8 (2.4%)	17 (5.1%)	97.7%
Phenice 1969	‐	218	7	108 (32.4%)	0	96.9%
Long bones	0.50	317	16	‐	0	95.2%
	0.70	301	10	‐	22 (6.6%)	96.8%
	0.88	259	5	‐	69 (20.7%)	98.1%

^a^ Excluded due to insufficient preservation of the pelvic bone; method not applicable.

^b^ Indeterminate; method applied but classification not assigned.

#### 3.2.2 Long bone LDA model

Performance showed clear threshold dependency. At 0.50 threshold, the model classified all individuals with 95.2% accuracy (317/333 correct, 16 misclassifications). Increasing the threshold to 0.70 improved accuracy to 96.8% (301/311 correct, 10 errors, 22 indeterminate or 6.6% indeterminate classifications). The conservative 0.88 threshold achieved optimal accuracy of 98.1% (259/264 correct, 5 errors, 69 indeterminate or 20.7% indeterminate classifications). Complete results are presented in Table 4.

#### 3.2.3 Sex‐specific performance

The addition of Tables 5 and 6 provides method performance by biological sex. For males (Table 5), the Bruzek and Phenice methods both maintained excellent sensitivity (≥99%), whereas the long bone model displayed progressive improvement from 92.6% at the 0.50 threshold to 97.7% at 0.88. Among females (Table 6), morphoscopic methods showed slightly lower accuracy (Bruzek = 95.4%; Phenice = 94.5%), while the LDA model demonstrated stable and high specificity across thresholds (97.7%–98.5%). Notably, class discrimination bias remained consistently equal or lower than the threshold of 5% [5] and increasing the classification threshold progressively reduced the class discrimination bias between male sensitivity and female specificity, yielding a more balanced performance across sexes.

**TABLE 5:** Results of sex estimation methods for the 161 males.

Male ‐ sensitivity						
Method	Threshold	Correct (*n*)	Incorrect (*n*)	Excluded^a^ (%)	Indeterminate (%)^b^	Accuracy (%)
Bruzek 2002	‐	155	0	2 (1.2%)	4 (2.5%)	100%
Phenice 1969	‐	115	1	45 (28%)	0	99.1%
Long bones	0.50	149	102	‐	0	92.6%
	0.70	144	7	‐	10 (6.2%)	95.4%
	0.88	126	3	‐	32 (19.9%)	97.7%

^a^ Excluded due to insufficient preservation of the pelvic bone; method not applicable.

^b^ Indeterminate; method applied but classification not assigned.

**TABLE 6:** Results of sex estimation methods for the 172 females.

Female ‐ specificity						
Method	Threshold	Correct (*n*)	Incorrect (*n*)	Excluded^a^ (%)	Indeterminate (%)^b^	Accuracy (%)
Bruzek 2002	‐	146	7	6 (3.5%)	13 (7.8%)	95.4%
Phenice 1969	‐	103	6	63 (36.6%)	0	94.5%
Long bones	0.50	168	4	‐	0	97.7%
	0.70	157	3	‐	12 (6.98%)	98.1%
	0.88	133	2	‐	37 (21.5%)	98.5%

^a^ Excluded due to insufficient preservation of the pelvic bone; method not applicable.

^b^ Indeterminate; method applied but classification not assigned.

As the difference between sensitivity and specificity remained equal or below 5%, subsequent analyses considered the overall accuracy without distinguishing between sexes. This approach reflects the practical equivalence in classification performance and facilitates direct comparison between methods in the following sections.

### 3.3 Agreement analysis and complementarity assessment

Cross‐method comparison revealed high concordance rates with method‐specific error patterns (Table 7). Bruzek‐Phenice agreement reached 95.4% (206/216 cases), and in the few cases of disagreement, each method correctly classified half of the individuals (5 cases each). Long bone model agreement with morphoscopic methods improved with increasing thresholds: Bruzek‐LDA agreement progressed from 92.9% (0.50 threshold) to 95.9% (0.88 threshold), while Phenice‐LDA agreement increased from 91.1% to 94.5%. Critically, when methods agreed, accuracy was 100%, indicating high reliability of consensus decisions. In other words, morphoscopic and metric methods never both misclassify biological sex at the same time. When disagreement occurred, misclassification patterns varied depending on the threshold used. Specifically, at 0.50, the morphological approach tended to outperform the metric model, with correct classification rates of 68% vs. 32% for Bruzek 2002, and 65% vs. 35% for Phenice (1969), respectively. As the posterior probability threshold increased, correct agreements remained perfect (100%), and in disagreement cases, accuracies corrected towards balanced distribution (with 50:50 for Bruzek‐LDA and 40:60 for Phenice‐LDA).

**TABLE 7:** Rate of agreement between morphoscopic and metric methods for estimating sex according to different classification thresholds.

Threshold	Method	Total compared	Accuracy % (*n*)	Agreement % (*n*)	Correct agreement % (*n*)	Correct disagreement % (*n*)
	Bruzek 2002 ‐Phenice 1969	216	B: 97.7 (211/216) P: 97.7 (211/216)	95.4 (206/216)	100 (206/206)	B: 50 (5/10) P: 50 (5/10)
0.50	Bruzek 2002 ‐Long bones	308	B: 97.8 (301/308) LB: 95.1 (293/308)	92.9 (286/308)	100 (286/286)	B: 68 (15/22) LB: 32 (7/22)
	Phenice 1969 ‐Long bones	225	P: 96.9 (218/225) LB: 94.2 (212/225)	91.1 (205/225)	100 (205/205)	P: 65 (13/20) LB: 35 (7/20)
0.70	Bruzek 2002 ‐Long bones	288	B: 97.9 (282/288) LB: 98.2 (279/288)	94.8 (273/288)	100 (273/273)	B: 60 (9/15) LB: 40 (6/15)
	Phenice 1969 ‐Long bones	209	P: 96.7 (202/209) LB: 96.2 (201/209)	92.8 (194/209)	100 (194/194)	P: 50 (8/15) LB: 50 (7/15)
0.88	Bruzek 2002 ‐Long bones	246	B: 98 (241/246) LB: 98 (241/246)	95.9 (236/246)	100 (236/236)	B: 50 (5/10) LB: 50 (5/10)
	Phenice 1969 ‐Long bones	181	P: 96.7 (175/181) LB: 97.8 (177/181)	94.5 (171/181)	100 (171/171)	P: 40 (4/10) LB: 60 (6/10)

*Note*: Agreement: identically sex estimation. Correct agreement: of these agreements, how many correctly predict sex? Correct desagreement: of the desagreement, which method is right? B: Bruzek 2002 [4]. P: Phenice 1969 [3]. LB: Long bones.

### 3.4 Population‐specific validation results

The study provides the first comprehensive population‐specific validation for contemporary Italian remains. The long bone LDA model demonstrated robust performance across the Italian sample, with accuracy levels comparable to recent machine learning studies on European populations [8, 17]. Threshold‐dependent performance offers operational flexibility for forensic practitioners balancing accuracy requirements against sample coverage needs.

Missing data analysis revealed that among 16 incorrectly classified individuals using LDA, 6 cases involved imputed values, highlighting the importance of complete measurement sets for optimal performance.

## 4 DISCUSSION

### 4.1 Methodological innovation and forensic applications

This study establishes the first direct comparison between morphoscopic pelvic methods and multivariable long bone analysis within a contemporary Italian population, addressing critical gaps in population‐specific validation [15, 16]. The threshold‐adjustable LDA framework introduces operational flexibility previously unavailable in forensic sex estimation, allowing practitioners to optimize the balance between classification certainty and sample coverage based on case‐specific requirements.

The observed performance levels align with recent advances in forensic anthropology, where machine learning approaches consistently achieve 90–97% accuracy in cross‐validation studies [8, 9, 10]. Additionally, our direct comparison highlights the practical complementarity of morphoscopic and metric approaches. Misclassification patterns showed minimal overlap between methods, suggesting that each may capture partially different aspects of sexual dimorphism. Nonetheless, further targeted analyses would be required to explore this interpretation in depth.

### 4.2 Population specificity and cross‐method validation

The population‐specific validation using the CAL Milano Cemetery Skeletal Collection addresses fundamental concerns about method transferability across demographic groups [17, 18]. Italian‐specific accuracy benchmarks established in this study (95.2%–98.0% for long bones, 96.9%–97.7% for morphoscopic methods) provide critical reference standards for forensic practitioners working with Mediterranean populations.

The finding that disagreement patterns show minimal overlap between methods supports the hypothesis that morphoscopic and metric approaches capture different aspects of sexual dimorphism. This complementarity suggests that combined methodological approaches may enhance overall classification reliability, particularly in challenging forensic contexts.

### 4.3 Operational advantages and practical implementation

Long bone metric methods offer several operational advantages when compared with morphoscopic approaches, particularly in forensic contexts characterized by fragmentation and the need for reproducible decision‐making. First, long bones are frequently preserved even when the pelvis is absent or incomplete, extending applicability to a wider range of cases. Second, while standardized scoring systems also exist for morphoscopic traits, metric measurements rely on continuous variables and internationally established osteometric protocols, which facilitate quantitative reproducibility and limit inter‐observer subjectivity.

A key distinction of the present approach lies in the integration of threshold‐adjustable posterior probabilities within a single multivariable framework, allowing practitioners to explicitly control the trade‐off between classification certainty and sample coverage. Although confidence levels can also be indirectly adjusted in morphoscopic methods through conservative scoring or trait selection, such adjustments are typically qualitative and method dependent. In contrast, the long bone LDA framework provides a transparent and numerically defined mechanism for threshold calibration, which can be consistently applied and audited across laboratories.

The demonstrated measurement reliability (rTEM <5%, ICC >0.90) supports the suitability of long bone metrics for forensic applications requiring high precision and traceability. Moreover, integration with existing forensic protocols requires minimal additional training, as the measurements follow established osteometric standards [29, 30], and the analytical workflow is fully reproducible.

### 4.4 Limitations and future directions

Some limitations warrant consideration. The present study shows that long bone LDA offers considerable advantages for sex estimation and increasing posterior probability threshold does increase the overall accuracy of sex classification. However, one should not overlook the trade‐off of these improvements, which is represented by the increase of the indeterminate classification rate of individuals (reaching 20.7% with the 0.88 threshold).

The contemporary Italian sample may not generalize to other populations or temporal periods, necessitating additional validation studies. Missing data imputation, while statistically sound, introduces uncertainty that may affect individual case reliability. The threshold selection, though empirically optimized, reflects sample‐specific characteristics requiring validation across diverse forensic contexts.

Moreover, although the Phenice 1969 method was adopted here for reasons of laboratory standard operating procedures and consistency with the Bruzek 2002 approach, we recognize that the updated Klales et al. [21] included in the MorphoPASSE method [7] could offer additional advantages. In particular, its use of scoring could provide a more nuanced framework for comparing morphological and metric results. Incorporating this approach in future analyses would therefore represent a promising aspect to refine and expand the results presented in this study.

Future research should examine: (1) integration with geometric morphometric approaches for enhanced precision, (2) machine learning ensemble methods combining morphoscopic and metric data, (3) validation against DNA‐confirmed forensic cases, and (4) development of population‐specific calibration protocols for diverse demographic groups.

### 4.5 Integration with contemporary forensic practice

These findings support the integration of long bone sex estimation as a complementary tool in forensic identification protocols. While morphoscopic pelvic assessment remains the primary method when preservation permits, long bones provide a reliable alternative for fragmentary remains. The threshold‐adjustable framework enables adaptation to varying operational requirements, from conservative forensic applications demanding high certainty to archaeological contexts prioritizing sample coverage.

The demonstrated complementarity between methods suggests that consensus‐based approaches, where multiple methods agree, provide enhanced confidence in sex estimation. Disagreement patterns may indicate cases requiring additional investigation or alternative identification approaches.

## 5 CONCLUSION

This study presents the first direct comparison between morphoscopic pelvic methods and metric long‐bone analyses for sex estimation in a contemporary Italian sample. Results show that misclassification overlap between the two approaches is limited, indicating that they provide complementary information and support the recommendation of an integrated approach in forensic casework.

This study validates long bone LDA as a reliable alternative to morphoscopic pelvic methods for sex estimation in contemporary Italian forensic contexts. The achieved accuracy levels (95.2%–98.5%) approach those of traditional morphoscopic methods while offering universal applicability and objective reproducibility.

The threshold‐adjustable probabilistic framework provides operational flexibility for balancing classification certainty against sample coverage requirements. Crucially, the demonstration of method complementarity rather than redundancy supports integrated approaches combining morphoscopic and metric methodologies for enhanced forensic reliability.

These findings establish population‐specific performance benchmarks for contemporary Italian forensic applications while contributing to the growing evidence base for machine learning integration in forensic anthropology. The methodology provides a framework for population‐specific validation studies essential for responsible implementation of sex estimation methods across diverse forensic contexts.

## CONFLICT OF INTEREST STATEMENT

The authors declare no competing interests.

## ETHICS STATEMENT

All research procedures received institutional ethical approval and followed current European guidelines for research ethics on human remains.

## Data Availability

The data that support the findings of this study are available from the corresponding author upon reasonable request.
